# Cisplatin +/− rucaparib after preoperative chemotherapy in patients with triple-negative or BRCA mutated breast cancer

**DOI:** 10.1038/s41523-021-00240-w

**Published:** 2021-03-22

**Authors:** Maitri Kalra, Yan Tong, David R. Jones, Tom Walsh, Michael A. Danso, Cynthia X. Ma, Paula Silverman, Mary-Claire King, Sunil S. Badve, Susan M. Perkins, Kathy D. Miller

**Affiliations:** 1 Indiana University Melvin and Bren Simon Cancer Center, Indianapolis, IN USA; 2 Department of Biostatistics, Indiana University School of Medicine, Indianapolis, IN USA; 3 University of Washington, Seattle, WA USA; 4 Virginia Oncology Associates/US Oncology, Norfolk, VA USA; 5 Siteman Cancer Center, Washington University, St. Louis, MO USA; 6 University Hospitals Ireland Cancer Center, Case Comprehensive Cancer Center, Cleveland, OH USA

**Keywords:** Translational research, Breast cancer, Cancer genetics

## Abstract

Patients with triple-negative breast cancer (TNBC) who have residual disease after neoadjuvant therapy have a high risk of recurrence. We tested the impact of DNA-damaging chemotherapy alone or with PARP inhibition in this high-risk population. Patients with TNBC or deleterious BRCA mutation (TNBC/BRCAmut) who had >2 cm of invasive disease in the breast or persistent lymph node (LN) involvement after neoadjuvant therapy were assigned 1:1 to cisplatin alone or with rucaparib. Germline mutations were identified with BROCA analysis. The primary endpoint was 2-year disease-free survival (DFS) with 80% power to detect an HR 0.5. From Feb 2010 to May 2013, 128 patients were enrolled. Median tumor size at surgery was 1.9 cm (0–11.5 cm) with 1 (0–38) involved LN; median Residual Cancer Burden (RCB) score was 2.6. Six patients had known deleterious *BRCA1* or *BRCA2* mutations at study entry, but BROCA identified deleterious mutations in 22% of patients with available samples. Toxicity was similar in both arms. Despite frequent dose reductions (21% of patients) and delays (43.8% of patients), 73% of patients completed planned cisplatin. Rucaparib exposure was limited with median concentration 275 (82–4694) ng/mL post-infusion on day 3. The addition of rucaparib to cisplatin did not increase 2-year DFS (54.2% cisplatin vs. 64.1% cisplatin + rucaparib; *P* = 0.29). In the high-risk post preoperative TNBC/BRCAmut setting, the addition of low-dose rucaparib did not improve 2-year DFS or increase the toxicity of cisplatin. Genetic testing was underutilized in this high-risk population.

## Introduction

Triple-negative breast cancer (TNBC) lacks expression of estrogen (ER), progesterone (PR), and human epidermal growth factor receptor-2 (HER2) receptors, accounts for ~15–20 percent of all breast cancers, and is more aggressive than other subtypes^1^. Though not synonymous, TNBC is classified as basal-like breast cancer via cDNA microarray ~75% of the time^2,3^. Approximately 75–80% of breast cancers that develop in women harboring deleterious *BRCA1* mutations have a triple-negative phenotype and basal-like gene expression. The correlation between TNBC, basal-like breast cancer, and *BRCA1* mutations suggests a similar underlying molecular pathogenesis that could be exploited therapeutically.

Inhibition of poly(ADP-ribose) polymerase (PARP) leads to impaired single-strand break (SSB) repair, ultimately leading to double-strand breaks (DSB) in replicative cells^4,5^. In *BRCA* wild-type cells, the DSBs are repaired via homologous recombination (HR), limiting damage. Conversely, in *BRCA*-deficient cells, HR is impaired and alternative pathways lead to complex rearrangements, loss of effective repair, and cell death (“synthetic lethality”). Clinical trials have demonstrated objective responses and/or an increased progression-free survival (PFS) with PARP inhibitor monotherapy in patients with germline *BRCA1* or *BRCA2* mutations^6–10^. The same *BRCA*-deficient defect in HR increases sensitivity to DNA-damaging chemotherapy compared to microtubule-acting agents. For example, response and PFS were superior with carboplatin compared to docetaxel in patients with mutated *BRCA* but not in patients with TNBC and intact *BRCA*^11^.

Neoadjuvant chemotherapy not only increases breast conservation but also provides an in vivo chemosensitivity assay, offering an ideal platform for clinical research. Although many will experience tumor shrinkage, only about ~25–45% of patients with TNBC will achieve a pathologic complete response (pCR), defined as the lack of invasive disease in the breast and regional lymph nodes at the time of surgery, with chemotherapy alone. Long-term follow-up of neoadjuvant studies consistently demonstrates significantly improved survival in patients who achieve a pCR compared to those with residual disease^12^, particularly in those with TNBC^13,14^. Those with substantial residual TNBC (Miller–Payne classification 1 or 2^15^ or RCB classification II or III^16^) have an inferior prognosis with only 35–40% remaining free of recurrence at 2 years. This high-risk group represents both an unmet medical need and an opportunity to accelerate the testing of novel agents in the early disease setting.

Based on the hypothesis that TNBC has an exploitable DNA damage repair defect similar to that seen in patients with the germline BRCA mutation, we undertook this randomized phase II trial to determine efficacy and safety of cisplatin alone or with rucaparib in patients with TNBC/BRCAmut who had substantial residual disease after standard neoadjuvant chemotherapy.

## Results

### Patient population

**Fig. 1:**
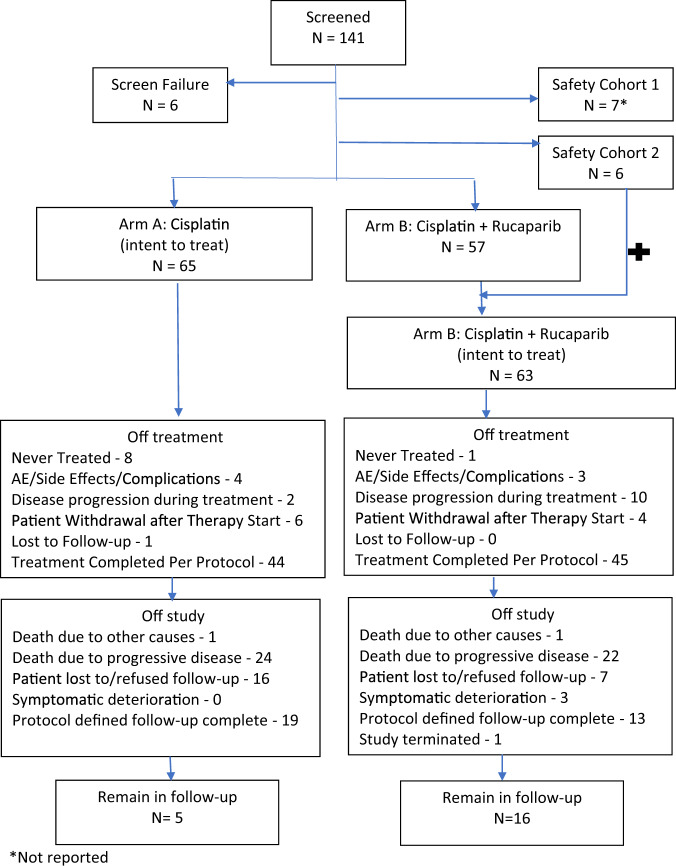
Consort diagram detailing the disposition of all patients enrolled. Patients enrolled in the safety cohort 1 are not reported in detail. Patients enrolled in safety cohort 2 are combined with patients randomized to Arm B for all intent-to-treat analyses.

From Feb 2010 to May 2013, 128 patients (intent-to-treat population) were enrolled in safety cohort 2 (*n* = 6) and the randomized portion (*n* = 122) of the study (Fig. 1). Patient characteristics are detailed in Table 1. Treatment arms were generally well balanced with a median age of 47.5 years (range 21–75 years), median residual tumor 1.9 cm, and involvement of one regional lymph node. The median RCB classification (graded as described in ref. ^16^) was 2.6 in cisplatin alone arm and 2.7 in the cisplatin plus rucaparib arm (median RCB score 53 vs. 59). Three patients in each arm had weak ER + tumors. Anthracycline exposure was more frequent in patients randomized to cisplatin (66.2% vs. 47.6%) but nearly all patients had received a taxane. Few patients had received carboplatin.

**Table 1:** Patients characteristics.

		Cisplatin (*N* = 65)		Cisplatin + rucaparib^a^ (*N* = 63)	
		*N*/value	%	*N*/value	%
Race	White	49	75.4	46	73
	Black or African American	13	20	11	17.5
	Asian	1	1.5	3	4.8
	Not reported/unknown	2	3.1	3	4.8
Ethnicity	Hispanic or Latino	6	9.2	3	4.8
	Non-Hispanic	57	87.7	53	84.1
	Not reported/unknown	2	3.1	7	11.1
Age	Median	48		47	
	Range	27–69		21–75	
ECOG PS	0	52	80	52	82.5
	1	13	20	11	17.5
Tumor size at diagnosis	Median	2.8 cm		3.9 cm	
	Range	1.2–7.0		1.3–5.0	
ER	Negative	61	93.8	60	95.2
	Positive	3	4.6	3	4.8
	Unknown	1	1.5	0	0
PR	Negative	62	95.4	60	95.2
	Positive	2	3.1	3	4.8
	Unknown	1	1.5	0	0
HER2	Negative	64	98.5	62	98.4
	Positive			1	1.6
	Unknown	1	1.5	0	0
*Germline mutation known prior to entry*					
BRCA1	No	64	98.5	62	98.4
	Yes	1	1.5	1	1.6
BRCA2	No	63	96.9	61	96.8
	Yes	2	3.1	2	3.2
*Neoadjuvant chemotherapy*					
Anthracycline	Yes	43	66.2	30	47.6
	No	22	33.8	33	52.4
Taxanes	Yes	60	92.3	56	88.9
	No	5	7.7	7	11.1
Carboplatin	Yes	0	0	6	9.5
	No	65	100	57	90.5
Bevacizumab	Yes	1	1.5	0	0
	No	64	98.5	63	92.1
*Residual disease*					
Tumor size at surgery (cm)^b^	*N*	54		57	
	Median	1.9 cm		1.9 cm	
	Range	0–9.0		0–11.5	
LN involved post-neoadjuvant chemotherapy^c^		*N* = 57		*N* = 60	
	Median	1		1	
	Range	0–15		0–38	
RCB^d^		*N* = 53		*N* = 59	
	Median	2.6		2.7	
	Range	0–5.0		0–5.3	
	SD	1.2		1.2	

^a^Includes patients in safety cohort 2 (*n* = 6) and all patients randomized to Arm B (*n* = 57). Characteristics from patients in safety cohort 1 are not reported.

^b^Tumor size could not be accurately measured in 11 patients.

^c^Eleven patients underwent sentinel node biopsy prior to neoadjuvant chemotherapy and did not have additional nodes resected at the time of definitive surgery.

^d^RCB could not be calculated in 16 patients.

### Efficacy

**Fig. 2:**
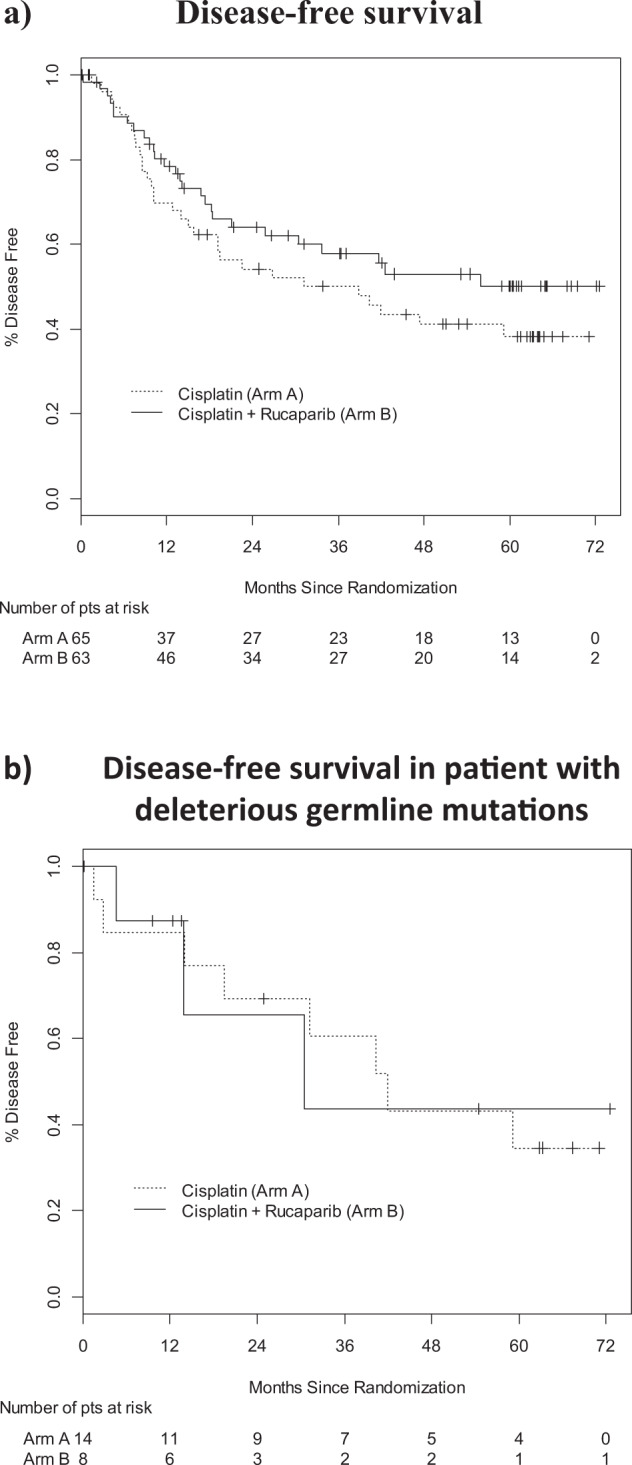
Disease-free survival. **a** All patients in the intent-to-treat analysis. Median DFS (38.8 months vs. not reached, *P* = 0.24) and 5-year DFS (38.3% vs. 50.1%; *P* = 0.24) were similar in both treatment groups. **b** Patients with deleterious germline *BRCA1* or *BRCA2* mutations (*n* = 22). Median DFS (19.5 vs. 13.8 months, *P* = 0.99) and 5-year DFS (34.6% vs. 43.8%; *P* = 0.24) were similar in both treatment groups.

The addition of rucaparib to cisplatin did not increase 2-year DFS (cisplatin 54.2% (95% CI: 39.8%, 66.6%) vs. cisplatin + rucaparib 64.1% (95% CI: 50.3%, 75.0%); *P* = 0.29) or 5-year DFS (38.3% (95% CI: 24.6%, 51.8%) vs. 50.1% (95% CI: 35.5%, 63.0%); *P* = 0.25, Fig. 2a).

### Drug exposure and toxicity

Overall more than two-thirds of patients completed the planned four cycles of cisplatin (69.2% cisplatin, 77.8% cisplatin + rucaparib), and median cisplatin dose intensity was similar in both arms (73.1 mg/m^2^ vs. 73.5 mg/m^2^). Cisplatin dose reduction due to toxicity, most commonly myelosuppression, was similar in both groups (18.5% vs. 23.8%). Only one patient in each arm reported grade 4 neutropenia (Table 2). In contrast, only 50.8% of the patients completed the planned 24 weeks of rucaparib maintenance. Eleven patients declined to start maintenance therapy, often due to the cost of frequent travel to the study site or a desire for early reconstruction. The most common reason for early discontinuation in patients starting maintenance therapy was disease progression (*n* = 10). Rucaparib dose was reduced in eight patients, four (6.3%) due to toxicity and four (6.3%) due to dosing error. Rucaparib dose delays were common (76.2%); 31 patients (49.2%) required rucaparib dose delays due to toxicity, most frequently due to fatigue; 25.4% due to scheduling constraints (holiday clinical closure, travel, etc.).

**Table 2:** Toxicity.

	Cisplatin (*N* = 65) *N* (%)			Cisplatin + rucaparib (*N* = 63)^a^ *N* (%)		
**NCI-CTC grade**	**2**	**3**	**4**	**2**	**3**	**4**
Neutropenia	16 (25)	11 (17)	0	13 (21)	16 (25)	1 (2)
Neutropenic fever	1 (2)	0	0	1 (2)	0	0
Anemia	5 (8)	0	0	7 (11)	0	0
Thrombocytopenia	2 (3)	0	0	0	2 (3)	0
Nausea	13 (20)	0	0	16 (25)	3 (5)	0
Anorexia	3 (5)	0	0	7 (11)	0	0
Fatigue	14 (22)	3 (5)	1 (2)	11 (17)	6 (10)	0
Tinnitus	16 (25)	1 (2)	0	12 (19)	1 (2)	0
Nephropathy	1 (2)	0	0	2 (3)	0	0
Neuropathy	1 (2)	0	0	3 (5)	1 (2)	0
Vomiting	5 (8)	0	0	7 (11)	3 (5)	0
Hepatic abnormality	0	1 (2)	1 (2)	0	3 (5)	0
Rash	2 (3)	0	0	2 (3)	0	0
Headache	2 (3)	0	0	5 (8)	0	0
Dysgeusia	3 (5)	0	0	1 (2)	0	0

Worst toxicity per patient according to NCI-CTC v 3.0.

^a^Includes toxicity reported during rucaparib maintenance.

Pharmacokinetic data were available from 60 patients treated with rucaparib (6 from safety cohort 2 and 54 randomized to cisplatin + rucaparib). Based on the in vitro IC90 against the target, adjusted for protein binding, a sustained plasma concentration of >5.9 ng/mL was projected to be needed in humans to inhibit PARP. Rucaparib exposure was quite variable with median concentration 282 ng/mL (range 82–2403 mg/mL) post-infusion on day 3. Of the 52 patients who received maintenance rucaparib, 16 received only IV rucaparib, 8 began IV and switched to oral when the oral formulation became available, and 28 received only oral rucaparib maintenance. During IV maintenance, the pre-dose sample was below the LLOQ (0.1 ng/ml) for rucaparib in 82% at week 1 and 37.5% at week 5. During oral maintenance, pre-dose rucaparib was below the LLOQ in 70% at week 1 and 23% at week 5.

### Germline genetics

Only six patients were known to have deleterious *BRCA* mutations (two *BRCA1* and four *BRCA2*) prior to study entry. Germline DNA, available in 101 consenting patients, was analyzed for the presence of deleterious mutations in *BRCA1* and *BRCA2*, as well as 21 other genes known to increase the risk of breast cancer (BROCA)^17,18^. This testing identified germline mutations in 22 patients, including 8 *BRCA1*, 12 *BRCA2*, and 2 *BRIP*1. DFS results were similar in the subset of 22 patients with deleterious mutations (2-year DFS for cisplatin 69.2% (95% CI: 37.3%, 87.2%) vs. 65.6% (95% CI: 15.7, 90.9%) for cisplatin + rucaparib; *P* = 0.88, Fig. 2b). Combining treatment arms, we found no difference in outcome in patients with versus without deleterious *BRCA* mutations (2-year DFS 68.6% (95% CI: 42.6%, 84.6%) cisplatin vs. 58.4% (95% CI: 46.4%, 68.7%) cisplatin + rucaparib; *P* = 0.44, 5-year DFS 36.4% (95% CI: 14.6%, 58.7%) cisplatin vs. 47.4% (95% CI: 34.9%, 58.9%) cisplatin + rucaparib, *P* = 0.41).

## Discussion

The high risk of recurrence in patients with TNBC/BRCAmut who have residual disease after neoadjuvant therapy provides a robust clinical laboratory for testing new therapies. We hypothesized that a DNA damage repair defect would lead to benefit from cisplatin, and that the addition of the PARP inhibitor rucaparib would amplify that benefit. This is a negative trial—the addition of low-dose rucaparib did not improve 2-year DFS. Unfortunately, our study had several limitations that leave the original hypothesis largely unanswered.

First, we lacked an appropriate contemporary control arm. Our study was conducted well before the results of the CreateX trial which suggested benefit for adjuvant capecitabine in this patient population^19^. Randomization to a “no adjuvant therapy” control was considered but ultimately rejected. Provider surveys and practice pattern data suggested that nearly half of all patients eligible for our trial received adjuvant therapy in the absence of supportive data and with a variety of different regimens, limiting acceptance of a no-treatment control arm and complicating analysis. As we hypothesized a favorable interaction between cisplatin and rucaprib, a two-by-two factorial design was not appropriate. A three-arm trial would have been scientifically superior but the sample size was prohibitive. Recognizing that would result would not be definitive, we compensated for the lack of control by selecting a high-risk population and targeting a large improvement in efficacy.

Second, neoadjuvant chemotherapy was not standardized increasing the heterogeneity of our patient population. Some may be surprised that only half of the patients enrolled in our trial received anthracycline-based neoadjuvant therapy. The use of anthracyclines in early-stage breast cancer was influenced by a prominent national debate with well-respected leaders arguing against and for use of anthracycline-based regimens, leading to wide regional variation in the use of anthracyclines. That variation is reflected by our patient population. Similarly, we allowed patients who had received carboplatin in the neoadjuvant setting to enroll, potentially limiting the benefit of cisplatin-based therapy. Our major interest was in the potential synergy of the combination, which we hypothesized may overcome resistance to DNA-damaging chemotherapy alone. As few patients in our trial had received neoadjuvant carboplatin, their impact is minimal.

Third, 2-year DFS was better in our patients than we had predicted, reducing the power of our study. Several factors may have contributed to this improved outcome. Over time patients with the less extensive disease were treated in the neoadjuvant setting. As the initial disease burden did not impact eligibility, we did not collect the data on the clinical stage at presentation to evaluate the impact of this shift in practice. We also included patients with a pCR in the breast and residual nodal disease. Several of those patients had the only microscopic residual nodal disease and would have been classified as RCB I with an excellent prognosis. Finally, in the absence of a control arm, we can not deduce or exclude the potential benefits from cisplatin monotherapy.

Finally, the enthusiasm for PARP inhibitors a decade ago, spurred by the high-profile presentation of data from a phase II trial of the putative PARP inhibitor iniparib^20^, accelerated protocol development. We rushed to activation while the initial dose-finding studies of rucaparib were ongoing and before the final oral formulation was available. While our design protected patient safety, we did not fully consider the potential negative implications of early activation. In this case, the dose of rucaparib used in our study (100 mg orally once a week) is substantially less than the recommended phase II dose (600 mg orally twice daily)^21,22^ currently used for maintenance therapy in patients with recurrent ovarian cancer. Our pharmacokinetic analysis further emphasizes the low dosage used in our trial, as more than a quarter of the patients had levels below the lower limit of detection with both intravenous and oral formulations. While rucaparib concentrations during combined therapy were above the level predicted to inhibit PARP in humans (>5.9 ng/mL), sustained PARP inhibition was almost certainly not achieved. The relative lack of myelosuppression, a pharmacodynamic hallmark of effective PARP inhibition, is consistent with the limited rucaparib exposure. Thus it is unlikely that the doses we used were sufficient to meaningfully inhibit PARP, leaving our initial hypothesis largely unanswered.

Despite these limitations, our trial has several important findings. First, germline genetic testing was, and based on recent reports by others^23–26^ remains, woefully underused. National Comprehensive Cancer Network (NCCN) guidelines recommend that all patients younger than 60 years with TNBC be offered genetic testing irrespective of family history^25^. With a median age of 47 years, over half of our patients would have met NCCN testing criteria, yet only six patients were known to have deleterious mutations at study entry. Fully two-thirds of the BRCA mutations identified by BROCA analysis were not identified during routine clinical care. We did not expect such sparse use of genetic testing and thus did not collect the data to determine if testing was not offered, not accepted, or not performed to due financial constraints. Given the impact of identifying a germline mutation, enhanced education^26^, and ongoing clinical vigilance to identify patients meeting testing guidelines is clearly needed. As a substantial proportion of women with breast cancer carrying germline pathogenic variants do not qualify for testing by NCCN criteria, expansion of current testing guidelines should be considered^27^.

In addition, biologic specimens collected in our study have yielded other important insights^28,29^. Analysis of matched somatic genomes pre-/post-neoadjuvant chemotherapy revealed the chaotic acquisition of copy gains and losses, including amplification of prominent oncogenes. Gene expression data revealed depletion of immune signaling, which was corroborated by decreases in tumor-infiltrating lymphocytes in post-neoadjuvant samples. We also found enrichment of actionable regulators of stem cell-like behavior and a poor prognosis associated with somatic gains in 18q, likely driven by putative upregulation of TGFß signaling through the signal transducer SMAD2.

Recognizing that TNBC is heterogenous at the genomic level^30–32^, a single therapeutic approach is less likely to be successful. Consequently, we designed a follow-up study to exploit targetable alterations found by sequencing residual disease. BRE12-158 (NCT02101385) randomized 196 TNBC patients with residual disease after neoadjuvant therapy to genomically directed therapy versus treatment of physician choice (capecitabine recommended based on the CreateX trial data). PFS results are expected within the next 12–18 months. Two ongoing national trials evaluate the impact of other therapeutic approaches. EA1131 (NCT02445391) follows our original hypothesis, comparing adjuvant platinum therapy (cisplatin or carboplatin) to capecitabine. Though all patients with TNBC and at least 1 cm of residual disease in the breast are eligible, the primary endpoint focuses on those with basal-like breast cancer. In contrast, S1418 (NCT02954874) evaluates the immune checkpoint inhibitor pembrolizumab, hoping to reverse the negative impact of immune depletion seen in residual disease specimens. Both trials are actively enrolling patients and include robust sample collection to support translational science.

## Methods

### Eligibility

Patients with Stage II-III TNBC/BRCAmut who had undergone neoadjuvant chemotherapy with an anthracycline and/or taxane-containing regimen were eligible if they had the substantial residual disease at the time of definitive surgical resection. We defined substantial residual disease as any one of the following: Miller–Payne class 1 or 2, RCB II or III, at least 2 cm of invasive disease in the breast, or persistent involvement of at least one axillary lymph node. Neoadjuvant cisplatin was prohibited but the incorporation of carboplatin as a component of neoadjuvant therapy was allowed. ER status was determined by local pathology assessment; patients with low levels of ER and/or PR expression (Allred Score ≤ 2 or <5% weak staining) were allowed. Patients with known deleterious *BRCA1* or *BRCA2* mutations were eligible with any degree of ER expression, but patients with HER2+ tumors were not eligible regardless of *BRCA* status. Patients who had breast-conserving surgery received adjuvant whole-breast radiation therapy. Postmastectomy radiotherapy was required for patients with primary tumor ≥5 cm or involvement of four or more lymph nodes. Postmastectomy radiation in other situations was left to the treating physician’s discretion. All radiation therapy was completed prior to study entry. Patients were required to have an ECOG PS 0 or 1 with adequate renal, bone marrow, and cardiac function. The protocol was approved by the Institutional Review Board, and all patients gave written informed consent prior to study enrollment.

### Treatment plan

As there was no prior experience with cisplatin and rucaparib in combination, the study began with two sequential safety cohorts to exclude prohibitive toxicity (Fig. 1). All patients received cisplatin 75 mg/m^2^ on day 1 every 21 days for four cycles. Cohort 1 (*n* = 7) received rucaparib 16 mg IV on days 1–3 in cycle 1, and in the absence of dose-limiting toxicity (DLT), 24 mg IV on days 1–3 in cycles 2–4. Cohort 2 (*n* = 6) received rucaparib 24 mg IV on days 1–3 in cycle 1, and in the absence of DLT, 30 mg IV on days 1–3 in cycles 2–4 (considered “full” dose rucaparib at the time). Patients in the safety cohorts were evaluated clinically and with laboratory assessments weekly during cycles 1 and 2. Cohort 2 began only after ≤1 of six patients in cohort 1 experienced DLT during the first two cycles of therapy. After ≤1 of six patients in cohort 2 experienced DLT during cycles 1 and 2, subsequent patients were randomized to one of two treatment arms. Arm A received cisplatin monotherapy. Arm B received cisplatin + rucaparib (24 mg IV for cycle 1, 30 mg IV for cycles 2–4 as in safety cohort 2) on days 1–3 every 21 days for four cycles followed by maintenance rucaparib for 24 weeks (30 mg IV once weekly). An oral rucaparib formulation became available when accrual was ~70% complete. At that point, the study was amended to use the oral formulation (100 mg once weekly, predicted to yield similar rucaparib exposure as 30 mg IV once weekly) during maintenance therapy only; all patients received the IV formulation for cycles 1–4. Optimal supportive care including antiemetics, hydration, antibiotics, and blood transfusion was permitted as clinically indicated and according to institutional guidelines. Prophylactic use of white blood cell growth factors was not allowed unless used in accordance with ASCO guidelines.

Toxicity was assessed based on the National Cancer Institute Common Toxicity Criteria (NCI-CTC) version 3.0. Dose modifications were based on nadir blood counts and interval toxicity, considering each drug individually. Cisplatin dose was reduced 25% for neutropenic fever, platelets less than 75,000/mm^3^, neutrophil count less than 1500/mm^3^ for >1 week, creatinine more than 1.5 times upper limit of normal (ULN), and grade 3 or more hepatic toxicity. Rucaparib dose was not adjusted for the first episode of neutropenic fever, reduced by 25% for a second episode, and discontinued for a third episode. Rucaparib dose was held and then resumed at the same dose upon recovery for neutrophil count <1500/mm^3^ and platelet count less than 100,000/mm^3^. Rucaparib was discontinued for any other grade 3 or 4 toxicity lasting >3 weeks. Upon completion of protocol therapy, patients were followed clinically every 4 months throughout years 1–2, then every 6 months for years 3–5. Annual breast imaging was required in all patients with remaining native breast tissue; consistent with NCCN^33^ and ASCO guidelines^34^, no other imaging or laboratory assessments were required during follow-up.

### Correlative studies

Archived tumor samples from the definitive surgery were banked for future analyses; when available a sample from the pre-neoadjuvant chemotherapy diagnostic biopsy was retrieved and banked. Analyses of available paired (pre–post-neoadjuvant chemotherapy) tumor samples have been reported separately^28^.

Blood for limited pharmacokinetic (PK) analyses was obtained pre-dose and 5 min (+/−5 min) prior to the end of rucaparib infusion on cycle 1 day 3, cycle 2 day 3, and then weeks 1 and 5 of maintenance therapy. In patients switching from the IV to oral formulation during maintenance therapy, PK sampling was repeated 2 h post-dosing during the 1st and 5th week of oral therapy. Plasma rucaparib concentration was quantified using midazolam as the internal standard, liquid-liquid extraction, and HPLC-MS/MS (ABSciex3200, Applied Biosystems). Rucaparib and midazolam were separated by gradient mobile phase (acetonitrile: 0.1% formic acid) and HPLC using a C8 column (Restek 5 µm 150 × 4.6 mm). The Q1/Q3 transitions for rucaparib and midazolam were 324/293 and 326/291, respectively. The lower limit of quantification (LLOQ) was 0.1 ng/mL using 100 µL of plasma.

Consenting patients (*n* = 101) provided a sample for germline genetic testing. DNA extracted from blood was sequenced using BROCA, a targeted capture and massively parallel sequencing test developed at the University of Washington^17^. Twenty-three genes were analyzed for this trial, including *BRCA1, BRCA2, ATM, ATR, BAP1, BARD1, BRIP1/FANCJ, CDH1, CHEK1, CHEK2, FAM175A/ABRAXAS, FANCM, MRE11A, NBN, PALB2/FANCN, PTEN, RAD51B, RAD51C, RAD51D, RINT1, SLX4/FANCP, TP53*, and *XRCC2*. For each gene, the entire locus was sequenced, including all coding exons, complete 5′- and 3′-untranslated regions (UTRs), all introns (after removing Alu repeats), and 5–15 kb flanking transcription start and stop sites. Sequencing reads were aligned to human reference genome hg19. Variants were identified by GATK and Pindel after indel realignment and base quality recalibration. Single nucleotide variants and small insertion-deletion variants (indels) were identified. Copy number variants (CNVs) were identified using the in-house pipeline of the King lab^35^. Mutations potentially leading to splicing errors were included only if experimental validation of the effect had been previously shown using RNA isolated from patients’ blood. Missense mutations were included only if proven experimentally to be damaging.

### Sample size and statistical analyses

The primary endpoint was 2-year DFS, including all local–regional recurrences, distant recurrences, and deaths from any cause as DFS events. Secondary clinical objectives included safety, 1-year and 5-year DFS. We hoped to detect an improvement in 2-year DFS from 40% in the control arm^16^ to 63.2% in the rucaparib arm (corresponding to an HR = 0.5) with 80% power using a one-sided log-rank test at the 0.10 significance level. This design required 38 events. Assuming an accrual over 13–18 months and an exponential survival, 102 patients were required for the primary analysis. The total sample size was increased to 135 to accommodate the two safety cohorts (*n* = 13) and to account for drop-outs (estimated at 20%). Patients in safety cohort 2 were combined with patients randomized to Arm B for analysis; patients enrolled in cohort 1 are not reported in detail and were not included in efficacy analyses.

DFS was compared between the two treatment arms using an un-stratified Kaplan–Meier analysis with a log-rank test. A specific comparison of 2-year DFS was made using a two-sample test based on the complementary log–log transformation as suggested in Klein et al.^36^. Similar methods were used to compare 2-year DFS for patients with versus without germline *BRCA* mutations. Limited PK sampling was included to complement ongoing PK analyses in other rucaparib trials, and thus only descriptive analyses were planned. The archived tumor and germline DNA samples were used to explore potential correlates of recurrence and sensitivity to PARP inhibition.

The Hoosier Cancer Research Network (HCRN) compiled data summary reports for this trial and submitted these reports monthly to the lead investigator; quarterly data summaries were reviewed by the Indiana University Melvin and Bren Simon Cancer Center (IUSCC) Clinical Trial Monitoring Committee.

### Reporting summary

Further information on research design is available in the Nature Research Reporting Summary linked to this article.

## Supplementary information

Reporting Summary Checklist

## Data Availability

The data generated and analyzed during this study are described in the following data record: 10.6084/m9.figshare.14061683^37^. The data are not publicly available for the following reason: data contain information that could compromise research participant privacy. However, the data can be made available upon reasonable request to the corresponding author. A list of all data files generated as part of the related study is available as part of the data record in the file “Kalra_et_al_2021_underlying_data_availability.xlsx”.
