# Supplementary material for: Cisplatin +/− rucaparib after preoperative chemotherapy in patients with triple-negative or BRCA mutated breast cancer
Source: NPJ Breast Cancer. 2021 Mar 22;7:29. doi: 10.1038/s41523-021-00240-w (PMC7985189; doi:10.1038/s41523-021-00240-w)
Supplement: Supplementary file 1 — Reporting Summary Checklist [file 41523_2021_240_MOESM1_ESM.pdf]

nature research

Double-blind peer review submissions: write DBPR and your manuscript number here instead of author names.

Corresponding author(s): Kathy D Miller

Last updated by author(s): 2021-02-23

Reporting Summary

Nature Research wishes to improve the reproducibility of the work that we publish. This form provides structure for consistency and transparency in reporting. For further information on Nature Research policies, see our Editorial Policies and the Editorial Policy Checklist.

Statistics

For all statistical analyses, confirm that the following items are present in the figure legend, table legend, main text, or Methods section.

n/a	Confirmed	
☐	☒	The exact sample size (*n*) for each experimental group/condition, given as a discrete number and unit of measurement
☐	☒	A statement on whether measurements were taken from distinct samples or whether the same sample was measured repeatedly
☐	☒	The statistical test(s) used AND whether they are one- or two-sided<br>*Only common tests should be described solely by name; describe more complex techniques in the Methods section.*
☐	☒	A description of all covariates tested
☐	☒	A description of any assumptions or corrections, such as tests of normality and adjustment for multiple comparisons
☐	☒	A full description of the statistical parameters including central tendency (e.g. means) or other basic estimates (e.g. regression coefficient) AND variation (e.g. standard deviation) or associated estimates of uncertainty (e.g. confidence intervals)
☐	☒	For null hypothesis testing, the test statistic (e.g. *F*, *t*, *r*) with confidence intervals, effect sizes, degrees of freedom and *P* value noted<br>*Give P values as exact values whenever suitable.*
☒	☐	For Bayesian analysis, information on the choice of priors and Markov chain Monte Carlo settings
☒	☐	For hierarchical and complex designs, identification of the appropriate level for tests and full reporting of outcomes
☒	☐	Estimates of effect sizes (e.g. Cohen's *d*, Pearson's *r*), indicating how they were calculated

Our web collection on statistics for biologists contains articles on many of the points above.

Software and code

Policy information about availability of computer code

Data collection	Clinical data was collected using electronic case report forms created for this study. All data was housed in OnCore., a commercially available trial management platform commonly commonly used across NCI cancer centers. OnCore provides a secure, web-based, role- and permission-controlled platform for data collection and storage. Key data elements were independently source verified during study monitoring.
Data analysis	Analyses were performed using SAS Version 9.4 (Cary, NC)

For manuscripts utilizing custom algorithms or software that are central to the research but not yet described in published literature, software must be made available to editors and reviewers. We strongly encourage code deposition in a community repository (e.g. GitHub). See the Nature Research guidelines for submitting code & software for further information.

Data

Policy information about availability of data

All manuscripts must include a data availability statement. This statement should provide the following information, where applicable:

- Accession codes, unique identifiers, or web links for publicly available datasets
- A list of figures that have associated raw data
- A description of any restrictions on data availability

Data that support the findings of this study are maintained by the HCRN. Data an the full protocol are available from the corresponding author upon reasonable request.

April 2020

Field-specific reporting

Please select the one below that is the best fit for your research. If you are not sure, read the appropriate sections before making your selection.

☒ Life sciences ☐ Behavioural & social sciences ☐ Ecological, evolutionary & environmental sciences

For a reference copy of the document with all sections, see nature.com/documents/nr-reporting-summary-flat.pdf

Life sciences study design

All studies must disclose on these points even when the disclosure is negative.

Sample size	Sample size was based on an estimated 2 year DFS of 40% in the control arm. We targeted a HR 0.5 (corresponding to a 63.2% 2 year DFS) in the experimental arm with 80% power using a one-sided log rank test at the 01 significance level. Assuming accrual over 13-18 months and exponential survival 102 patients were required for the primary analysis. Total sample size was increased to allow for the planned safety run-in cohorts and for drop-outs.
Data exclusions	None
Replication	This is a single clinical trial and has not been replicated.
Randomization	Initial patients were assigned to safety cohort 1, then safety cohort 2. Once prohibitive toxicity was excluded, all subsequent patients were randomly assigned to cohort A or cohort B.
Blinding	There was no blinding

Reporting for specific materials, systems and methods

We require information from authors about some types of materials, experimental systems and methods used in many studies. Here, indicate whether each material, system or method listed is relevant to your study. If you are not sure if a list item applies to your research, read the appropriate section before selecting a response.

Materials & experimental systems

n/a	Involved in the study	
☒	☐	Antibodies
☒	☐	Eukaryotic cell lines
☒	☐	Palaeontology and archaeology
☒	☐	Animals and other organisms
☐	☒	Human research participants
☐	☒	Clinical data
☒	☐	Dual use research of concern

Methods

n/a	Involved in the study	
☒	☐	ChIP-seq
☒	☐	Flow cytometry
☒	☐	MRI-based neuroimaging

Human research participants

Policy information about studies involving human research participants

Population characteristics	Details can be found in Table 1 of the manuscript. All subjects were women, median age 47.5 years with a median residual tumor measuring 1.9 cm and involving 1 axillary node.
Recruitment	Subjects were recruited by participating investigators at 24 US sites.
Ethics oversight	Hoosier Cancer Research Network. IU Simon Cancer Center Data and Safety Monitoring Board.

Note that full information on the approval of the study protocol must also be provided in the manuscript.

Clinical data

Policy information about clinical studies

All manuscripts should comply with the ICMJE guidelines for publication of clinical research and a completed CONSORT checklist must be included with all submissions.

Clinical trial registration	NCT01074970
Study protocol	Full protocol available upon request from the corresponding author or to HCRN

Data collection	Recruitment from 2/2010 through 5/2013. Data was collected at 24 participating US sites and entered into OnCore. Key data elements were source verified during monitoring
Outcomes	Primary endpoint as 2-year DFS including all local-regional recurrences, distant recurrences and deaths from any cause as events. Secondary endpoints included safety, 1-year and 5-year DFS. Similar analyses were performed restricted to subjects carrying mutated breast cancer genes.
